# RIPK4 Suppresses the Invasion and Metastasis of Hepatocellular Carcinoma by Inhibiting the Phosphorylation of STAT3

**DOI:** 10.3389/fmolb.2021.654766

**Published:** 2021-06-18

**Authors:** Haoran Li, Dingan Luo, Lakshmi Huttad, Mao Zhang, Youpeng Wang, Juan Feng, Yunfeng Ding, Bing Han

**Affiliations:** ^1^ Department of Hepatobiliary and Pancreatic Surgery, The Affiliated Hospital of Qingdao University, Qingdao, China; ^2^ Asian Liver Center, Department of Surgery, Stanford University, Palo Alto, CA, United States

**Keywords:** signal transducer and activator of transcription, prognosis, metastasis, invasion, receptor interacting serine/threonine kinase 4, hepatocellular carcinoma

## Abstract

Receptor interacting serine/threonine kinase 4 (RIPK4) is a member of the threonine/serine protein kinase family; it plays related functions in a variety of tumours, but its biological function has not been fully revealed. It has been reported that it is differentially expressed in hepatocellular carcinoma (HCC). Our research aimed to reveal the role of RIPK4 in the progression of HCC and to reveal the biological behaviour of RIPK4 in HCC. We analysed the differences in RIPK4 expression in HCC by using a publicly available data set. By using PCR, Western blotting and immunohistochemical staining methods, we detected the expression level of RIPK4 in HCC patient specimens and studied the relationship between the expression of RIPK4 and the clinicopathological features of HCC patients. The prognostic data were combined to analyse the relationship between RIPK4 and HCC patient survival and tumour recurrence. We found that the expression level of RIPK4 in nontumour tissues was significantly higher than that in tumour tissues, and the level of RIPK4 was significantly positively correlated with postoperative survival and recurrence in HCC patients. Further, our study found that RIPK4 inhibits the progression of HCC by influencing the invasion and metastasis of HCC and that overexpression of RIPK4 reduces the invasion and metastasis of HCC by inhibiting epithelial-mesenchymal transition (EMT) and the STAT3 pathway. In *in vivo* experiments, overexpression of RIPK4 stably inhibited HCC metastasis. To summarize, our research revealed the relationship between RIPK4 and the prognosis of patients with HCC. We discovered that RIPK4 affects the invasion and metastasis of HCC through the EMT and STAT3 pathways. Targeted inhibition of the RIPK4 gene and the STAT3 pathway may be potential therapeutic strategies for inhibiting the postoperative recurrence and metastasis of HCC.

## Introduction

Primary liver cancer is one of the most common cancers in the world; it is the sixth most common cancer in the world, and the fourth most common cause of cancer-related death in the world (Freddie et al., 2018). Its morbidity and mortality rates remain high. Hepatocellular carcinoma (HCC) accounts for up to 90% of all primary liver malignancies and is a major international health problem (Grandhi et al., 2016). Despite recent advances in surgical and cancer drug treatment, the prognosis of patients is still not favourable (Yegin et al., 2016). The postoperative recurrence and metastasis of HCC patients are still the main reasons for the poor prognosis and high mortality rates of patients. To improve the prognosis/survival of HCC patients, it is very important to explore the molecular mechanism of the invasion and metastasis of HCC to identify new molecular regulatory targets.

Receptor interacting serine/threonine kinase 4 (RIPK4) was first discovered by yeast two-hybrid experiments. RIPK4 is a protein that is capable of interacting with protein kinase C (PKC) β1 and PKCδ (Chen et al., 2001), and the protein is located on chromosome 21q22.3. Its C-terminal region indicates that it is a member of the threonine/serine protein kinase family (Bähr et al., 2000). Increasing evidence shows that RIPK4 is inextricably linked to human diseases, especially tumours. Studies have shown that RIPK4 is upregulated in nasopharyngeal carcinoma tissues and facilitates the progression of tumours by promoting the expression of VEGF in nasopharyngeal carcinoma cells (Yongqian et al., 2018). Activation of the RAF1/MEK/ERK pathway is induced by PEBP1 degradation in pancreatic cancer and promotes the migration and invasion of pancreatic cancer cells (Qi et al., 2018). Studies have also shown that increased expression of RIPK4 makes the disease of patients with cervical squamous cell carcinoma progress rapidly and produces a poor prognosis (Liu et al., 2015). Interestingly, in addition to promoting tumour progression, other related studies have found that RIPK4 can also inhibit tumour progression; for example, RIPK4-mediated inhibition of STAT3 signalling slows the progression of lung cancer (Kopparam et al., 2017) and plays a role in inhibiting tumour progression in tongue squamous cell carcinoma and squamous cell carcinoma of the skin (Xinhua et al., 2014) (Chen et al., 2017). There is a current report on the role of RIPK4 in HCC (Heim et al., 2015). The study confirmed that RIPK4 plays an important role in the development of HCC as a tumour suppressor gene and elaborated on the differential expression of RIPK4 in HCC tissues and normal tissues. However, the report did not reveal detailed RIPK4 research data in HCC or the specific mechanism by which RIPK4 inhibits the development of HCC. Hence, further research could be performed to understand the role and related mechanisms of RIPK4 in the development and progression of HCC.

Signal transducer and activator of transcription 3 (STAT3) is a transcription factor that can regulate the occurrence and progression of cancer (Furtek et al., 2016). Studies have shown that upregulating STAT3 expression can promote the invasion and metastasis of colorectal cancer (Wang et al., 2019). STAT3 is also a key factor in mediating the invasion of nasopharyngeal carcinoma cells (Lin et al., 2017). STAT3 also promotes the progression of HCC (Wang et al., 2020) (Wang et al., 2017).

In our study, we examined the effect of RIPK4 on the invasion and metastasis of HCC, assessed its role in the epithelial-mesenchymal transition (EMT) of HCC, and explored the possible molecular mechanism by which RIPK4 regulates the invasion and metastasis of HCC. We evaluated the clinical significance of RIPK4 and indicated its potential as a new therapeutic target and prognostic marker for HCC.

## Materials and Methods

### Patients and Specimens

A total of 112 tumour tissues and adjacent liver tissues were collected from patients undergoing liver resection at the Affiliated Hospital of Qingdao University from January 2013 to December 2014. The patient samples and clinical data were obtained with written informed consent. The study was approved by the Ethics Committee of the Affiliated Hospital of Qingdao University, and all the protocols in our study were performed according to the related guidelines and regulations.

### Bioinformatics Analysis

We used the UALCAN online bioinformatic website (Chandrashekar et al., 2017) to search and analyse the mRNA expression of RIPK4 in normal and tumour tissues (http://ualcan.path.uab.edu/cgi-bin/ualcan-res.pl). Next, we used OncoLnc (Anaya, 2016) (http://www.oncolnc.org) to analyse the relationship between RIPK4 and the prognosis of HCC patients.

### Cell Culture and Reagents

The human HCC cell lines HCCLM3, Huh7, Hep3b, and HepG2 and the normal cell line LO-2 were purchased from the Type Culture Collection Committee of the Chinese Academy of Sciences (Shanghai, China). The cells were incubated in 10% foetal bovine serum (FBS; Gibco; Thermo Fisher Scientific) and Dulbecco’s modified Eagle’s medium (DMEM, HyClone; Thermo Fisher Scientific) supplemented with penicillin/streptomycin (100 U/ml) (Solarbio; Beijing, China). These cells were cultured in a humidified atmosphere of 95% air and 5% CO_2_ at 37°C.

Colivelin, an activator of the STAT3 pathway, was purchased from MCE (No. HY-P1061A; Shanghai, China).

### Lentivirus Transfection

We reconstructed the overexpression vector with the purchased plasmid. The lentiviral vector system was purchased from Genomeditech Biotechnology Co., Ltd. (Shanghai, China). Lentiviral infection was performed according to a previous research protocol (Zhang et al., 2019), the culture medium was replaced two days after infection, and 2 μg/ml puromycin (Solarbio, Beijing, China) was added for screening.

### Immunohistochemistry

Immunohistochemical staining of RIPK4 was performed on the specimens after obtaining tissue samples from patients. According to previous studies (Zhang et al., 2019), the IHC score of RIPK4 depends on the intensity of staining in cells and the percentage of positively stained cells. The staining intensity of the specimens was recorded as 0 (negative), 1 (low), 2 (medium) or 3 (high). Similarly, the degree of staining was recorded as 0 (0% staining), 1 (1–25% staining), 2 (26–50% staining) or 3 (51–100% staining). The total IHC score was determined by multiplying the intensity score by the degree of staining, ranging from 0 to 9. A final score below 4 (intensity score × percentage score) was considered to represent negative staining, and a score of 4 to 9 was considered to represent positive staining.

### Quantitative Reverse Transcription-Polymerase Chain Reaction (RT-qPCR)

Total RNA was extracted from the treated HCC cell lines using RNAiso Plus reagent (TaKaRa Biotechnology, Dalian, China), and single-stranded DNA (cDNA) was synthesized using the PrimeScript™ RT reagent Kit (TaKaRa Biotechnology, Dalian, China). RT-qPCR was performed using TB Green® Premix Ex Taq™ II (Tli RNaseH Plus). The PCR primers are listed in Supplementary Table S1. Three independent experiments were performed to standardize the results for each target to those of GAPDH levels, and the data are shown as the mean ± SD.

### Western Blot Analysis

Western blot analysis of protein expression was performed as previously described reference required (Zhang et al., 2019). Protein samples were separated on a sodium dodecyl sulfate-polyacrylamide gel electrophoresis (SDS-PAGE) gel (20 μg per lane). Protein bands were detected using United States Bio-Rad (ChemiDoc XRS+). The antibodies and dilution ratios used in this study are listed in Supplementary Table S1. ImageJ software was used to quantify the Western blotting results, and statistical analysis was performed accordingly.

### Wound Healing Assay

Lentivirus-infected cells were seeded in 6-well plates at 10 × 104 cells per well. The cells were allowed to adhere the surface, and once they reached 80% confluence, the monolayer was wounded using a 200-μl pipette tip to streak each hole vertically. The cells were washed with PBS 3 times and incubated in serum-free DMEM for another 36 h. Images of the scratches were captured with a microscope camera. Data were collected from three independent experiments. The percentage of wound healing was calculated using the following formula: [1- (empty area 24 (36) h/empty area 0 h)] × 100.

### Transwell Migration Assay

Transwell chambers with 8-µm pores (Corning, NY) were used to evaluate the migration and invasion of Huh7 and Hep3B cells. The upper chamber was filled with 5 × 104 cells from each sample in serum-free medium for migration analysis. We diluted Matrigel at a ratio of 1:3 to cover the bottom of the chamber in advance and then suspended 5 × 105 cells in serum-free medium in the upper chamber. After incubating the cells at 37°C for 24 h, the cells migrating to the bottom of the filter were incubated with 0.5% crystal violet at room temperature for 20 min. For quantitative measurement, five areas (at ×100 magnification) were randomly selected under an optical microscope, and the cells were counted.

### Animal Experiment

All experimental protocols involving animals were approved by the Ethics Committee of the Affiliated Hospital of Qingdao University. Female BALB/c nude mice (4–6 weeks old) were purchased from Beijing Life River Experimental Animal Technology Co., Ltd. (Beijing, China). The animals were divided into an experimental group and a control group, with four in each group. The mice were injected with 200 μL of 2 × 106 Huh7 Lv-RIPK4 cells or 2 × 106 Huh7 Lv-con suspension cells through the tail vein. During the experiment, the experimental group and the control group were treated in the same way. After 6 weeks, the mice were sacrificed, and the liver tissue was excised.

### Statistical Analysis

All statistical analyses were performed using SPSS 19.0 software or GraphPad Prism 7.0 software. The chi-square (χ^2^) test was used to analyse the classified data. Cox proportional hazard models were used for univariate and multivariate survival analysis. Data are expressed as the mean ± standard deviation (SD), and Student's t test or one-way or two-way ANOVA was used to compare the means of independent samples. The log rank test was used to compare Kaplan-Meier survival curves. *p* < 0.05 was considered statistically significant. R software was used to construct a nomogram model containing prognostic factors related to disease-free survival (DFS) and overall survival (OS) at 1, 3 and 5 years. Then, the discriminatory capabilities of the nomograms and other variables were assessed with receiver operating characteristic (ROC) curves, and the clinical utility of the nomograms and other variables was carefully investigated using decision curve analysis (DCA) to compensate for the limitations of the ROC curves.

## Results

### RIPK4 is Downregulated in HCC and Predicts a Poor Prognosis

**FIGURE 1:**
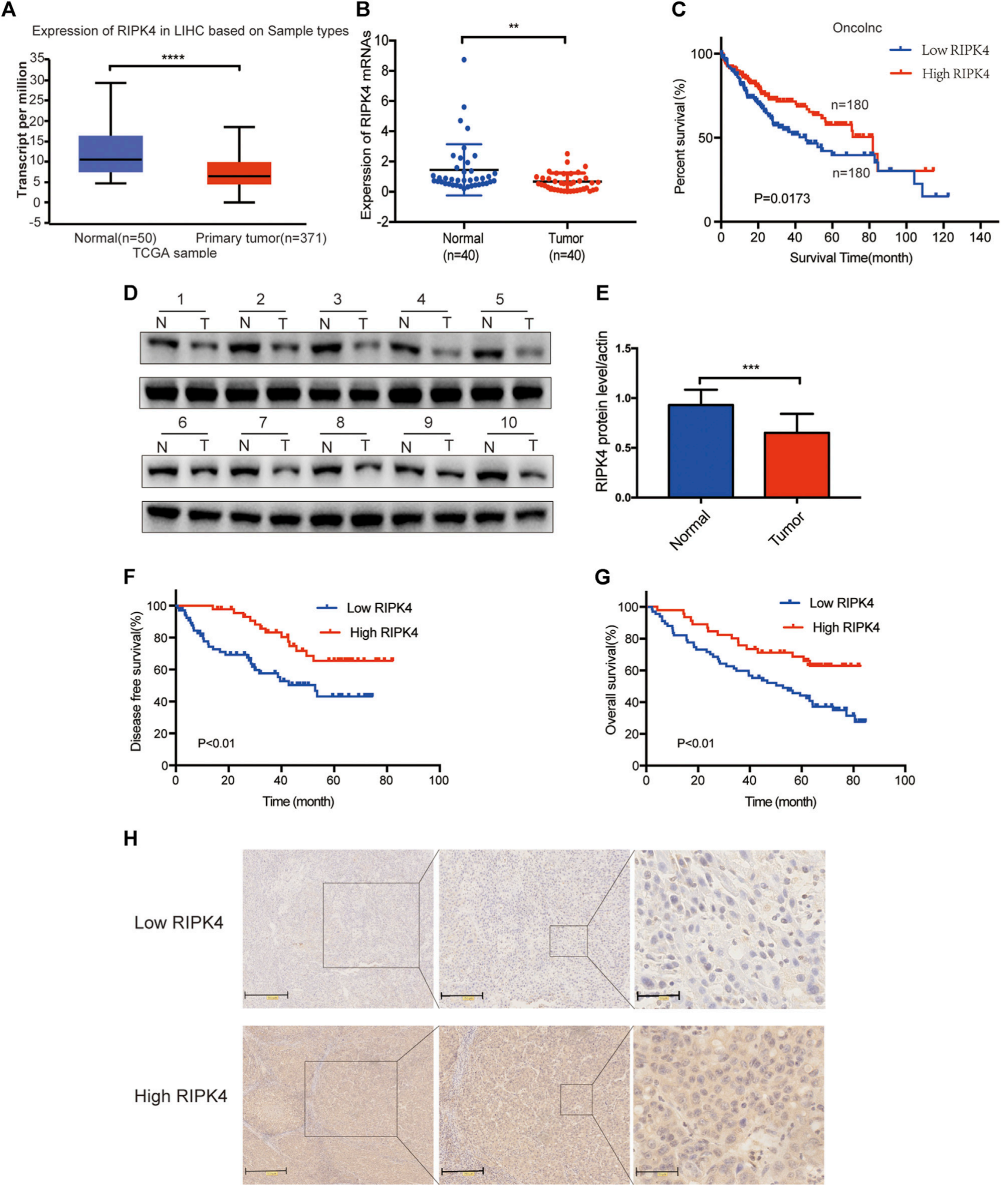
The expression of RIPK4 is reduced in HCC and is associated with poor prognosis. **(A)** We used ualcan data to analyze the expression level of RIPK4 mRNA in HCC and normal tissues. **(B)** Real-time quantitative PCR was used to analyze the expression levels of RIPK4 mRNA in the tumor tissues and adjacent tissues of 40 patients with HCC. **(C)** Correlation between RIPK4 mRNA expression of oncolnc database and overall survival in patients with HCC (*n* = 360). **(D)** Western blot of RIPK4 expression in tumor tissues and normal liver tissues. **(E)** RIPK4 expression levels were normalized according to *ß*-actin expression levels. **(F/G)** Kaplan-Meier plots of OS and DFS in HCC patients with higher and lower RIPK4 expression levels. **(H)** Representative images of RIPK4 immunohistochemical staining in HCC and normal liver tissue. **p < 0.01; ***p < 0.001; ****p < 0.0001.

In our study, we analysed the mRNA expression level of RIPK4 in HCC through the UALCAN web resource (http://ualcan.path.uab.edu/cgi-bin/ualcan-res.pl) and found that the mRNA expression level of RIPK4 was significantly reduced in HCC tissues vs. normal tissues (*p* < 0.0001) (Figure 1A). We performed RT-qPCR (*n* = 40) to detect RIPK4 mRNA expression in cancer tissues and adjacent normal liver tissues in patients with HCC, and the results revealed that RIPK4 mRNA expression was significantly reduced in cancer tissues compared with adjacent tissues (*p* < 0.05, Figure 1B). To further verify the clinical significance of RIPK4, we found that the average OS rate of patients with high RIPK4 mRNA expression was higher than that of patients with low RIPK4 mRNA expression through the OncoLnc data set (http://www.oncolnc.org) (Figure 1C). We examined the expression of RIPK4 by immunohistochemical staining of 112 HCC wax block sections created from patient samples. The results showed that the expression of RIPK4 in HCC tissue was significantly reduced compared to that in adjacent normal liver tissue, and the staining intensity of RIPK4 was also lower than that in adjacent normal liver tissue (Figure 1H). To clarify the clinical relevance of RIPK4, we examined the relationship between RIPK4 expression and clinicopathological characteristics in the 112 patients involved in our study. The analysis showed that high RIPK4 expression was negatively correlated with microvascular invasion (χ^2^ = 4.795, *p* < 0.05) (Table 1). Kaplan-Meier analysis of all 112 patients showed that the median DFS and median OS rates of patients with high RIPK4 expression were significantly higher than those of patients with low RIPK4 expression (Figure 1F/G). Finally, we assessed the prognostic value of RIPK4 in patients with HCC by univariate and multivariate Cox regression analysis. The results showed that high expression of RIPK4 was an independent risk factor for OS and DFS in patients with HCC (Table 2 and Table 3).

**TABLE 1:** Correlation of RIPK4 expression with clinicopathological features in HCC.

Parameters	Number	RIPK4		χ^2^	*P*
		Low	High		
Gender				0.015	0.903^a^
Male	94	56	38		
Female	18	11	7		
Age(y)				0.595	0.44^a^
<50	21	11	10		
≥50	91	56	35		
Alcohol abuse				0.134	0.715^a^
Yes	32	20	12		
No	80	47	33		
Tumor size (cm)				0.178	0.673^a^
≤5	62	31	19		
>5	50	36	26		
Tumor margin (cm)				0.303	0.582^a^
≤2	97	59	38		
>2	15	8	7		
Pathologic differentiation				1.202	0.273^a^
Middle and low	81	51	30		
High	31	16	15		
Microvascular invasion				4.795	***0.029*** ^a^
No	71	37	34		
Yes	41	30	11		
Liver cirrhosis				0.131	0.717^a^
No	23	13	10		
Yes	89	54	35		
HBV infection				1.000^c^
No	8	5	3		
Yes	104	62	42		
Capsule invasion				1.649	0.199^a^
No	37	19	18		
Yes	48	39	27		
Portal vein tumor thrombus				0.490	0.484^b^
No	101	62	39		
Yes	11	5	6		
AFP level (ng/L)				0.315	0.575^a^
≤400	78	48	30		
>400	34	19	15		
ALB level (g/L)				0.492	0.483^a^
≤35	19	10	9		
>35	93	57	36		
ALT level (U/L)				1.871	0.171^a^
≤60	87	55	32		
>60	25	12	13		
AST level (U/L)				2.332	0.127^a^
≤42	81	52	29	
>42	31	15	16	
PLT level (10^4^/μL)				0.967	0.326^a^
≤10	17	12	5	
>10	95	55	40	
TBIL level (μmol/L)				1.099	0.295^a^
≤22	90	56	34	
>22	22	11	11	

a Pearson’s χ^2^ test.

b Continuity correction by χ^2^ test.

c Fisher’s exact test; *p < 0.05, statistically significant.

The meaning of the bold values: *p* < 0.05

**TABLE 2:** Univariate and Multivariate analysis of variables with overall survival.

Variable	Univariate HR (95% CI)	*p* value	Multivariate HR (95% CI)	*p* value
Gender (male vs. female)	1.197 (0.606–2.366)	0.605		
Age (y, <50vs.≥50)	1.020 (0.517–2.012)	0.955		
Alcohol abuse	1.521 (0.628–2.449)	0.536		
Tumor size (cm, <5vs.≥5)	1.242 (0.746–2.066)	0.405		
Tumor margin (cm, <2vs. ≥2)	1.027 (0.487–2.165)	0.943		
Differentiation (Middle and low vs. High)	0.959 (0.541–1.700)	0.886		
Microvascular tumor thrombus (Yes vs.No)	1.839 (1.105–3.063)	0.019^a^	1.757 (1.053–2.931)	0.031^a^
Liver cirrhosis (No vs. Yes)	0.831 (0.448–1.544)	0.559		
Capsule invasion (Yes vs.No)	1.291 (0.735–2.266)	0.374		
Portal vein tumor thrombus (Yes vs.No)	2.163 (0.979–4.778)	0.056		
AFP level (ng/L, >400 vs. ≤400)	1.259 (0.735–2.155)	0.401		
ALB level (g/L, ≤35vs.>35)	0.833 (0.433–1.603)	0.585		
ALT level (U/L, ≤60vs.>60)	1.234 (0.688–2.215)	0.481		
AST level (U/L, ≤42vs.>42)	1.349 (0.782–2.325)	0.282		
PLT level (10^4^/μl, ≤10vs.>10)	0.702 (0.379–1.300)	0.261		
TBIL level (μmol/L, ≤22vs.>22)	1.339 (0.734–2.440)	0.341		
RIPK4(High vs. Low)	0.467 (0.263–0.830)	0.009^a^	0.485 (0.273–0.863)	0.014^a^

The meaning of the bold values: *p* < 0.05

HR, hazard ratio; CI, confidence interval.

a *p* < 0.05, statistically significant.

**TABLE 3:** Univariate and Multivariate analysis of variables with disease-free survival.

Variable	Univariate HR (95% CI)	*p* value	Multivariate HR (95% CI)	*p* value
Gender (male vs. female)	1.404 (0.552–3.569)	0.476		
Age (y, <50vs.≥50)	0.973 (0.432–2.191)	0.947		
Alcohol abuse	0.958 (0.482–1.903)	0.902		
Tumor size (cm, ≥5 vs. <5)	1.941 (1.062–3.544)	0.031^a^	1.883 (1.024–3.462)	0.042^a^
Tumor margin (cm, <5vs. ≥5)	0.490 (0.151–1.584)	0.233		
Differentiation (Middle and low vs. High)	1.009 (0.518–1.966)	0.979		
Microvascular tumor thrombus (Yes vs.No)	2.408 (1.316–4.407)	0.004^a^	1.898 (1.028–3.503)	0.041^a^
Liver cirrhosis (Yes vs.No)	2.862 (1.022–8.016)	0.045^a^		
Capsule invasion (Yes vs.No)	2.070 (1.040–4.118)	0.038*		
Portal vein tumor thrombus (Yes vs.No)	0.908 (0.281–2.938)	0.872		
AFP level (ng/L,≤400vs.>400)	1.106 (0.576–2.123)	0.762		
ALB level (g/L,≤35vs.>35)	0.666 (0.328–1.354)	0.262		
ALT level (U/L,≤60vs.>60)	0.909 (0.448–1.846)	0.792		
AST level (U/L,≤42vs.>42)	1.260 (0.665–2.386)	0.479		
PLT level (10^4^/μL,≤10vs.>10)	0.697 (0.322–1.506)	0.358		
TBIL level (μmol/L,≤22vs.>22)	0.832 (0.386–1.795)	0.640		
RIPK4(High vs. Low)	0.408 (0.212–0.785)	0.007^a^	0.409 (0.211–0.793)	0.008^a^

The meaning of the bold values: *p* < 0.05

HR, hazard ratio; CI, confidence interval.

a *p* < 0.05, statistically significant.

## Predictive Value of Nomograms Based on Differences in RIPK4 Expression Between Cancer and Adjacent Normal Tissues in Patients With HCC

**FIGURE 2:**
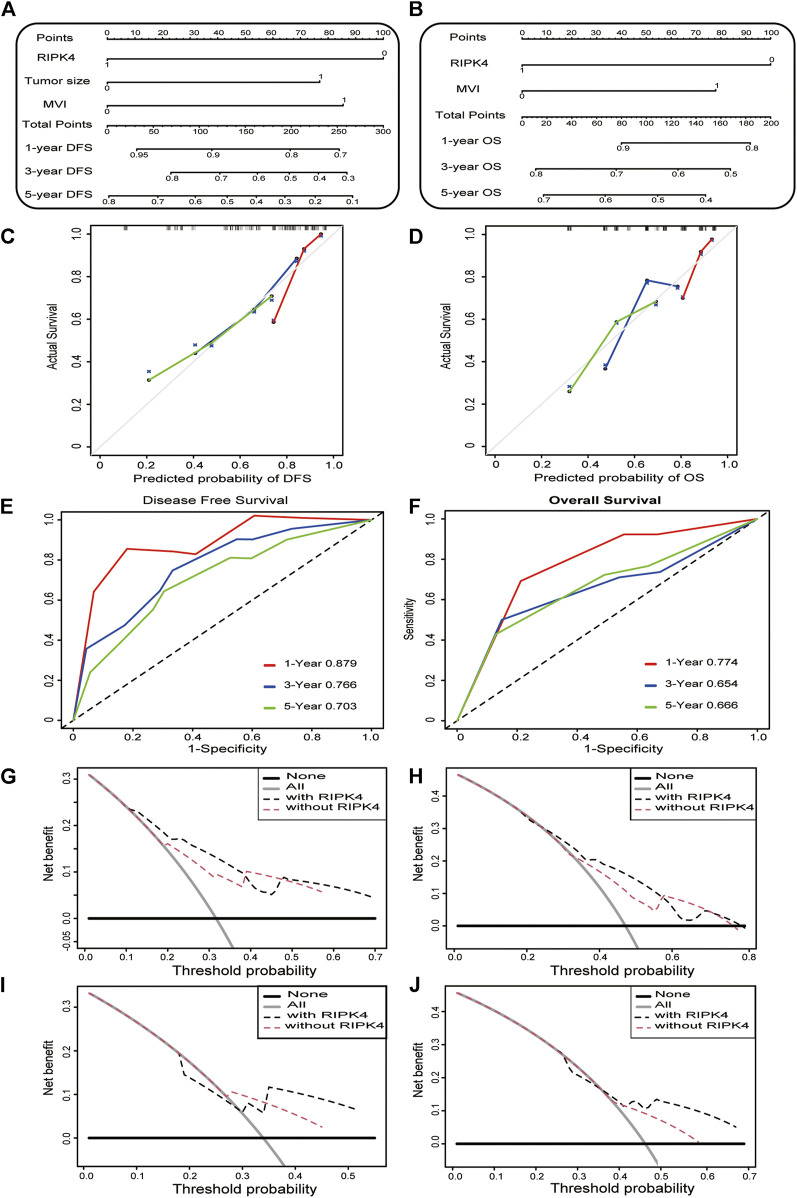
Construction and calibration of nomogram model. a-b. Nomogram developed to predict the incidence of DFS **(A)** and OS **(B)** in HCC at 1, 3 and 5 years **(C,D)** The calibration curve showing the predictive accuracy of the 1-year, 3-years, and 5-years DFS rate **(C)** and OS rate **(D)** of HCC patients in the study. **(E,F)** The ROC curve shows the 1, 3, and 5-years DFS **(left)** and OS **(right)** ratios in the study. **(G–J)** The DCA curve of the clinical utility of the 3-years **(G)** and 5-years DFS **(H)** nomogram in the study. DCA curve shows the clinical utility of the 3-years and **(I)** 5-years **(J)** OS nomogram in the study.

To provide clinically relevant quantitative methods to predict the incidence of DFS and OS at 1, 3 and 5 years, two nomogram models were established based on the multivariate analysis results of this study (Figure 2A/B). The nomograms were generated, and each independent risk factor was scored. The total scores were calculated, and the probability of individual recurrence and death was demonstrated by a vertical line based on the total score. In addition, the internally verified C index was used to calculate the prognostic accuracy of the DFS and OS models. The concordance indexes (C-indexes) of DFS and OS were 0.7398 (0.6618–0.8178) and 0.6791 (0.5819–0.7763), respectively, indicating that the nomogram models predicted approximately 73.98 and 67.91% probabilities of recurrence and death in individuals. This shows that our nomogram models have excellent predictive capabilities. The calibration curve in the internal verification explains the prediction accuracy for 1, 3 and 5-years DFS (Figure 2C) and OS (Figure 2D) rates. The calibration chart shows a high degree of agreement between the predictions for individual HCC recurrence and death and the actual observations. To further verify the superiority of our nomograms in assessing the prognosis of HCC patients, we drew the ROC curve of the nomogram models. The area under the curve (AUC) was used to evaluate the distinguishing ability of each model. Interestingly, the 1, 3 and 5-years AUCs of our nomograms reflect the high diagnostic value of our nomogram models (Figure 2E/F). We also used (clinical decision curve) DCA to evaluate the potential clinical effects of the nomograms with or without RIPK4 in this study. Our results show that the nomograms with RIPK4 showed excellent performance (Figures 2G–J). Our experimental results reveal that RIPK4 plays an indispensable role in these models by greatly enhancing its predictive performance.

### RIPK4 Inhibits the Migration and Invasion of HCC Cells

**FIGURE 3:**
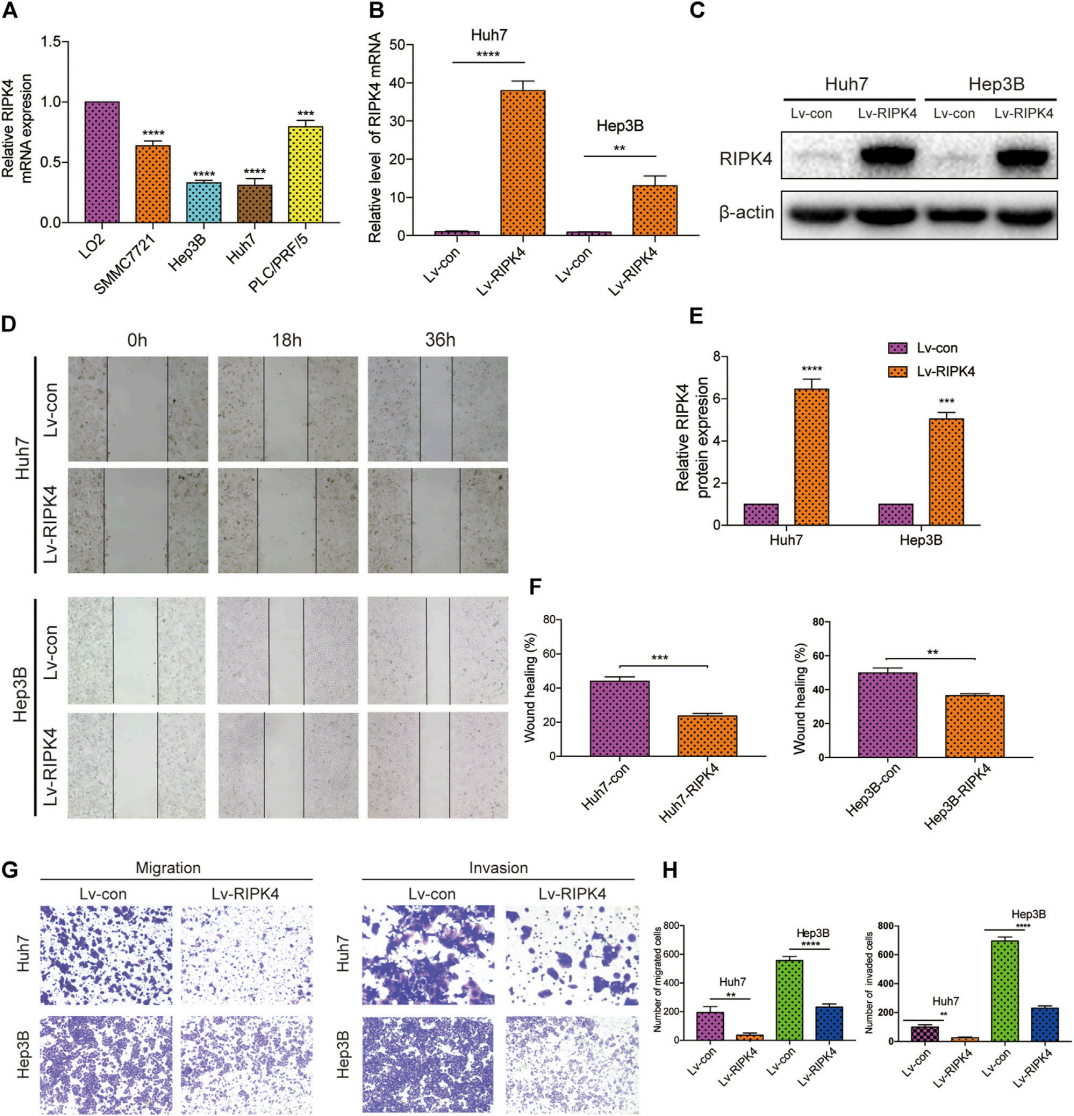
RIPK4 inhibits the migration and invasion of HCC cells. **(A)** Real-time quantitative PCR analysis of RIPK4 mRNA levels in HCC cells compared to L02 cells. **(B)** Real-time quantitative fluorescent PCR analysis of RIPK4 expression after lentivirus transfection. **(C)** Western blotting analysis of RIPK4 expression after lentivirus transfection, and *ß*-actin was used as a control. **(D)** The wound healing showed that the migrated ability of Huh7-RIPK4 and Hep3B-RIPK4 cells was decreased. **(E)** Statistical analysis of three independent data of western blotting experiments. **(F)** Statistical analysis of three independent data of wound healing experiments. **(G)** Representative images (100 times) of Huh7 and Hep3B cell line Transwell migration and Matrigel invasion experiments. **(H)** Statistical analysis on three independent data of Transwell experiment. All experiments were performed three times independently, and the data are expressed as mean ± SD. **p < 0.01; ***p < 0.001.

Before detecting the function of RIPK4 in various cell lines, we assessed the expression of RIPK4 in several HCC cell lines with different invasion and metastasis capabilities. Compared with normal liver L02 cells, the RIPK4 mRNA levels of the SMMC7721, Hep3B, Huh7, and PLC/PRF/5 cell lines were significantly lower than those of the L02 cell line, and the expression level of RIPK4 was lowest in the Hep3B and Huh7 cell lines (Figure 3A). We overexpressed RIPK4 in Hep3B and Huh7 cell lines through lentivirus infection experiments and detected high overexpression efficiency using RT-qPCR and Western blotting (Figures 3B,C). Further, we examined the effect of RIPK4 on the migration and invasion of HCC cells. Wound healing experiments showed that after overexpression of RIPK4 in Huh7 and Hep3B cell lines, the migration ability of cells was significantly reduced (Figure 3D). In the Transwell migration and Matrigel invasion experiments, the overexpression of RIPK4 also significantly reduced the migration and invasion of Huh7 and Hep3B cells (Figure 3G). These results initially suggest that RIPK4 is involved in inhibiting the invasion and metastasis of HCC.

### RIPK4 Inhibits EMT and the STAT3 Signalling Pathway

**FIGURE 4:**
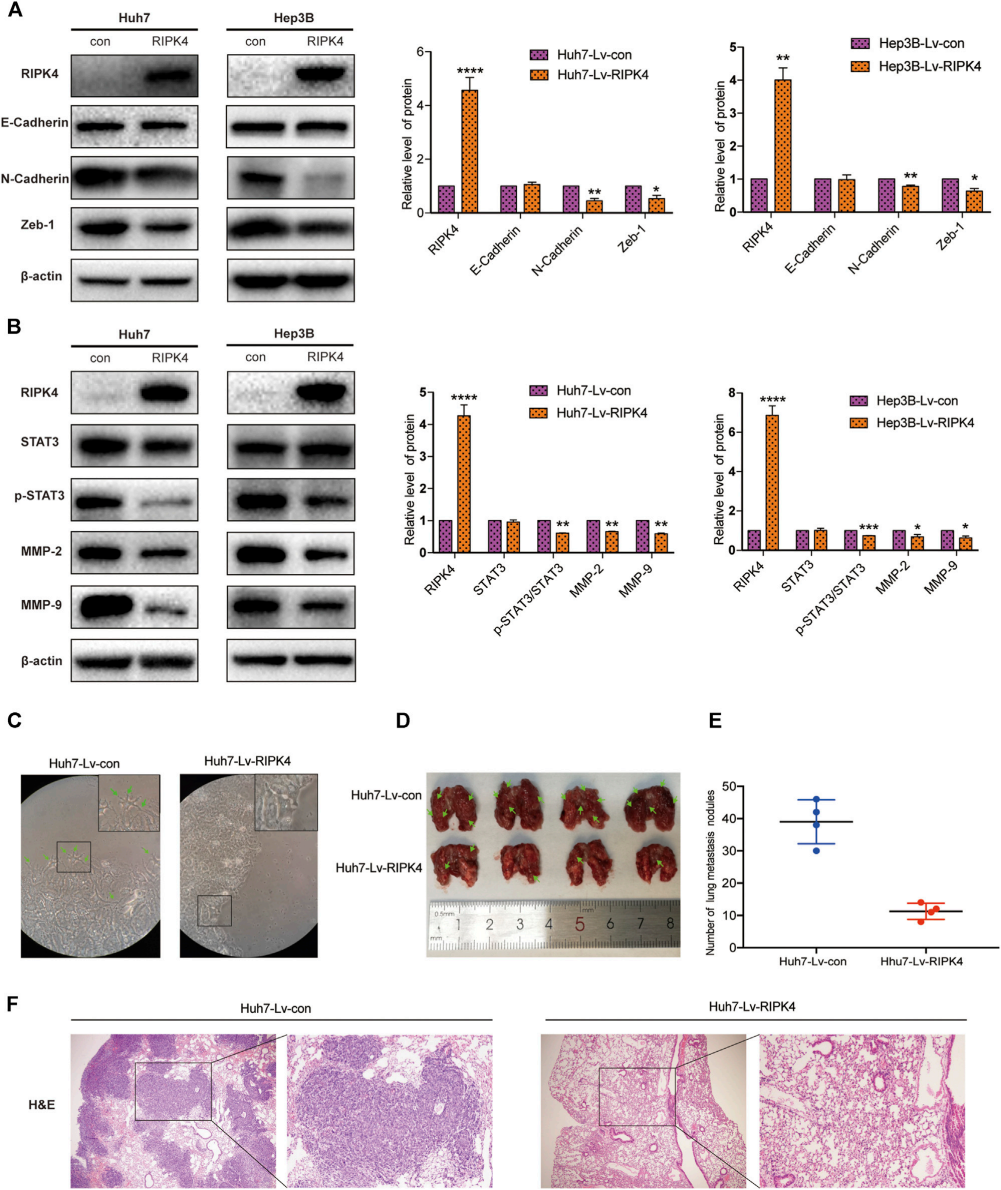
RIPK4 inhibits EMT progression and STAT3 pathway activation in HCC. **(A)** Western blotting analysis of the expression levels of E-Cadherin, N-Cadherin, Vimentin, Zeb-1 protein in Huh7 and Hep3B cells infected with Lv-con and Lv-RIPK4 lentivirus, and *ß*-actin was used as a control. **(B)** Western blotting analysis of STAT3, p-STAT3, MMP-2, MMP-9 protein expression levels in Huh7 and Hep3B cells infected with Lv-con and Lv-RIPK4 lentivirus, and *ß*-actin was used as a control. **(C)** Representative images of huh7 morphology with Lv-con and Lv-RIPK4 lentivirus. **(D,E)** Representative images of lung metastases in mice and statistical analysis of the number of metastases. **(F)** Representative H&E staining pictures of mouse lung metastatic nodules. The data are expressed as mean ± SD. **p < 0.01; ***p < 0.001.

EMT refers to the transformation of cells from an epithelial phenotype into a mesenchymal phenotype. In this process, the motility and invasiveness of epithelial cells are increased. We applied wound healing experiments and Transwell experiments to explore the relationship between RIPK4 and EMT in HCC. We used Western blotting to detect the expression levels of E-cadherin, N-cadherin and Zeb-1 in Huh7 and Hep3B cells in the control and overexpression groups. Western blotting data showed that the expression of E-cadherin, an epithelial cell marker, did not change significantly, while the expression of N-cadherin, vimentin and zinc finger protein (Zeb-1), mesenchymal cell markers, was significantly reduced after RIPK4 overexpression in Huh7 and Hep3B cells (Figure 4A). Because RIPK4 can strongly inhibit the migration and invasion of HCC cells over long distances, we next explored the role of RIPK4 in HCC and discussed the signalling pathway that regulates tumour metastasis. Western blotting analysis showed that RIPK4 overexpression reduced the protein levels of MMP-2 and MMP-9 in Huh7 and Hep3B cells, and the decrease in p-STAT3/STAT3 was statistically significant (Figure 4B). In addition, Huh7 cells infected with Lv-RIPK4 tended to exhibit a mesenchymal morphology. In contrast, the control Con-RIPK4-infected Huh7 cells had rounded borders, showing an epithelial morphology (Figure 4C).

### RIPK4 Inhibits the Metastasis of HCC by the STAT3 Signalling Pathway

**FIGURE 5:**
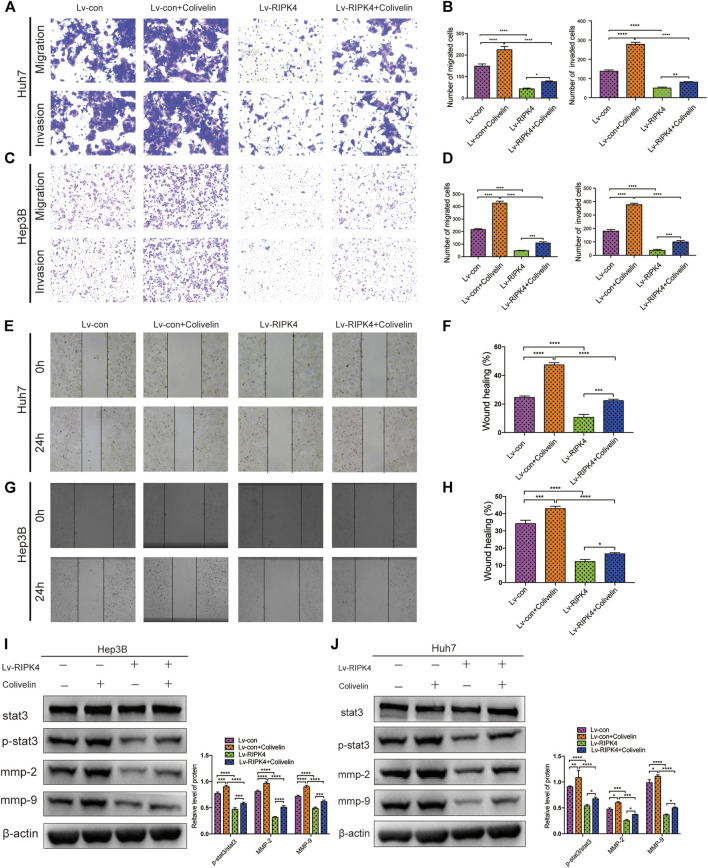
RIPK4 inhibits the metastasis of HCC by STAT3 signaling pathway. **(A,B)** Comparison of Huh7 cell migration and invasion ability of each group by transwell experiment. **(C,D)** Comparison of Hep3B cell migration and invasion ability of each group by transwell experiment. **(E,F)** Comparison of Huh7 cell ability of each group by Wound healing assay. **(G,H)** Comparison of Hep3B cell ability of each group using Wound healing assay. **(I)** Western blotting analysis of the expression of STAT3 and p-STAT3 and MMP9 and MMP2 protein. *ß*-actin was used as a internal control. **(J)** Western blotting analysis of the expression of STAT3 and p-STAT3 and MMP9 and MMP2 protein of Hep3B cell. *ß*-actin was used as a loading control. The data are expressed as mean ± SD. **p < 0.01; ***p < 0.001.

**FIGURE 6:**
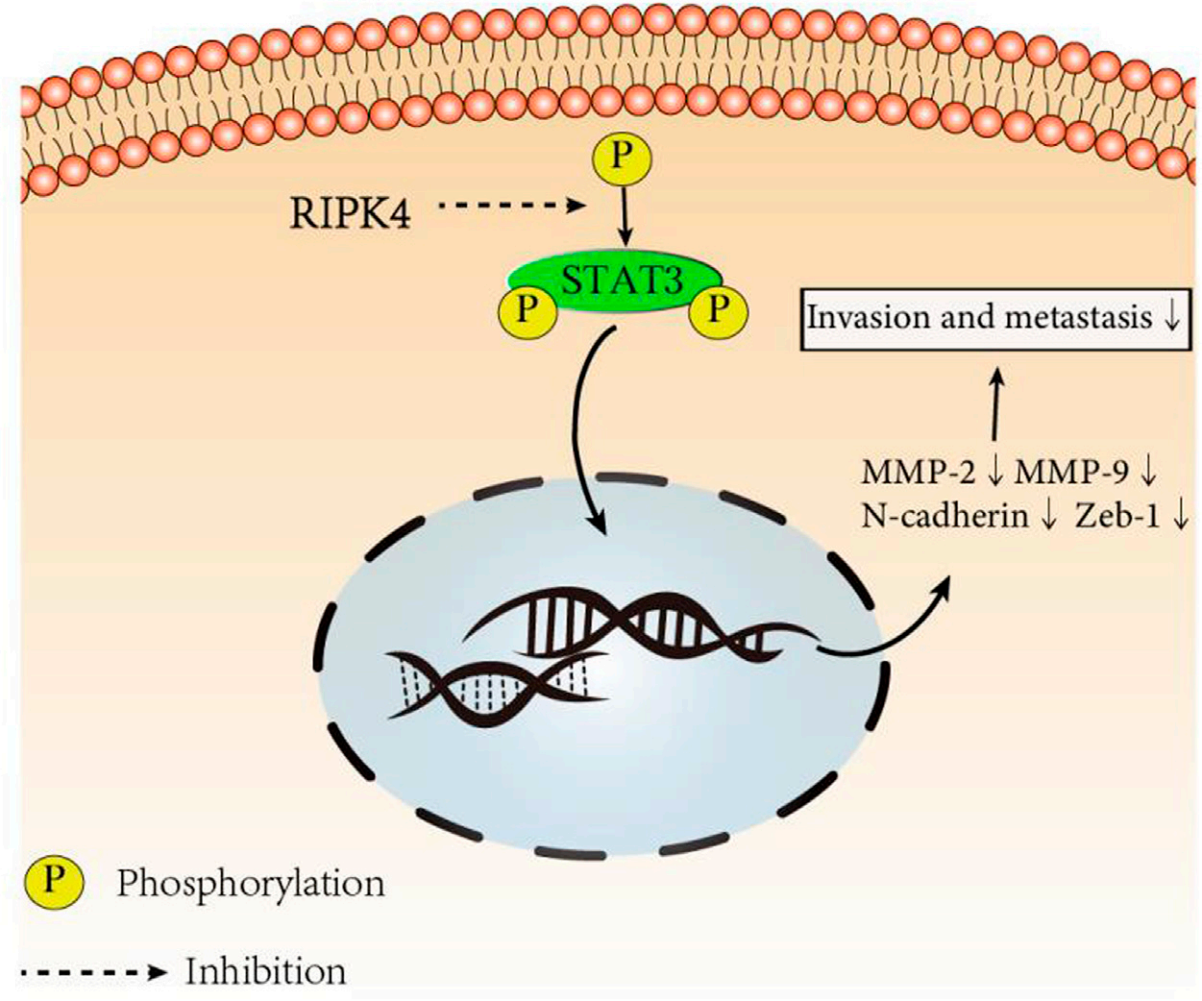
RIPK4 inhibits the invasion and metastasis of hepatocellular carcinoma by mediating the STAT3 signal pathway.

Since we discovered the relationship between RIPK4 and the STAT3 signalling pathway and its downstream molecular targets, we next further explored whether RIPK4 inhibits HCC invasion and metastasis through the STAT3 signalling pathway. According to previous studies, colivelin can activate the STAT3 pathway in human non-small-cell lung cancer cells and oesophageal cancer cells (Nyiramana et al., 2020) (Wen-Chin et al., 2016). Our research proved that colivelin promoted the migration and invasion of HCC cells (Figures 5A–H) and simultaneously increased MMP-2 and MMP-9 protein expression compared to that in control cells (Figures 5I,J). However, the migration and invasion of HCC cells treated with the combination of RIPK4 and colivelin were significantly reduced (Figures 5A–H), and the protein expression of MMP-2 and MMP-9 also decreased (Figures 5I,J). The migration and invasion ability was weaker (Figures 5A–H) and the protein expression of MMP-2 and MMP-9 was lower in HCC cells overexpressing RIPK4 than in other cell populations (Figures 5I,J). We proved that RIPK4 inhibits the invasion and metastasis of HCC cells through the STAT3 pathway (Figure 6).

### RIPK4 inhibits HCC Cell Metastasis *in vitro*

We further examined the effect of RIPK4 in HCC in mice. We injected Huh7-RIPK4 cells infected with lentivirus and their control cells through the tail vein of mice to observe whether RIPK4 can affect HCC *in vivo*. Six weeks after injection, we observed lung metastatic nodules and found that the Huh7-RIPK4 group had fewer and smaller lung metastatic nodules than the control group (Figures 4D,E). These results also further prove that RIPK4 participates in inhibiting the invasion and metastasis of HCC.

## Discussion

HCC is one of the most common malignant tumours worldwide and has high mortality and recurrence rates. It is important to study and understand the mechanism of HCC at the molecular level, as surgery and existing treatments have not been able to improve the cure rate of patients. In our attempt to understand the mechanism by which RIPK4 inhibits the STAT3 pathway, we utilized publicly available data sets and patient specimen data in our study to confirm the differential expression of RIPK4 in HCC and normal liver tissues. We explained that RIPK4 is an effective gene for inhibiting HCC metastasis and exerts a cancer suppressing effect by inhibiting the STAT3 pathway, as demonstrated by wound healing, Transwell and Western blotting experiments. Additionally, we found that RIPK4 can inhibit the progression of HCC by reversing the EMT process. In addition, *in vivo* experiments in mice confirmed that RIPK4 is a tumour suppressor gene.

RIPK4 is a threonine/serine protein kinase (Bähr et al., 2000). Some studies have indicated that RIPK4 expression is significantly lower in HCC tissues than in adjacent normal liver tissues (Heim et al., 2015). Our study confirmed that the expression of RIPK4 in HCC tumour tissues is significantly lower than that in normal liver tissue according to public data sets, and we applied tissue sample data collected from patient samples from our study to verify the expression of RIPK4 in HCC tumour tissues. At the mRNA and protein levels, the expression of RIPK4 in HCC tissue is significantly reduced compared with that in normal liver tissue, confirming previous research conclusions (Heim et al., 2015). We verified the differential expression of RIPK4 in HCC tumour tissues and normal tissues by PCR and Western blotting experiments. In addition, studies have reported that the expression of RIPK4 in cervical squamous cell carcinoma and pancreatic cancer is higher than that in normal tissues and that high expression of RIPK4 is related to a poor prognosis (Qi et al., 2018) (Liu et al., 2015). However, RIPK4 is found in well-differentiated tongue squamous cell carcinoma samples, and the level is significantly higher than that in poorly differentiated tongue squamous cell carcinoma samples (Xinhua et al., 2014). As of now, there is no report stating that RIPK4 affects the prognosis of HCC patients. Therefore, we used the public data set OncoLnc and an online prognostic website to predict the prognosis of HCC patients. In addition, we analysed the correlation between RIPK4 expression and clinicopathological factors in the patients involved in our study, and the results revealed that low RIPK4 expression was associated with microvascular invasion and that RIPK4 can be used as an independent risk factor for OS and DFS in patients with HCC. Patients with HCC expressing high levels of RIPK4 do have better OS and DFS than patients with HCC expressing low levels of RIPK4, which is also in accordance with the results from public database analysis.

In previous research on RIPK4, we found that RIPK4 affects the occurrence and development of different types of tumours in various ways, and there is a lack of research reports on RIPK4 in HCC. Thus far, we have confirmed the relation of RIPK4 with HCC through functional tests and confirmed that high expression of RIPK4 can inhibit the invasion and metastasis of HCC cells in culture systems and in mouse models. We designed the wound healing experiment and the Transwell experiment and found that HCC cells stably transfected with lentivirus had visibly migrated after 36 h of the scratch experiment. Moreover, the HCC cell invasion and metastasis abilities were assessed in serum-free medium that restricted the growth of HCC cells. For *in vivo* experiment, we established a mouse xenograft model. Mice overexpressing RIPK4 had fewer metastatic lung nodules than control mice. Our results indicate that RIPK4 plays an important role in inhibiting the invasion and metastasis of liver cancer.

Based on the results of functional tests, we studied the relationship between RIPK4 and EMT at the molecular level. Although E-cadherin, a surface marker of epithelial cells, can effectively reflect the tightness of contact between cells and the spread of tissue cells to distant organs and tissues (Giannelli et al., 2016) (Yang et al., 2018) (Gan et al., 2016), our research indicates that RIPK4 is not related to E-cadherin in HCC. In contrast to epithelial proteins, some mesenchymal proteins, such as N-cadherin and Zeb-1, have been reported to promote tumorigenesis and development (Zhou et al., 2017) (Hashiguchi et al., 2013) (Dong et al., 2016). Our research illustrates that RIPK4 can reduce the expression of N-cadherin, Zeb-1, and vimentin in HCC, and our research also found that RIPK4 can regulate HCC EMT and reduce the invasion and metastasis of HCC cells. The RIPK4-overexpressing hepatoma cell lines also showed more epithelial cell-like morphology than other cell groups. However, further research is needed to clarify the direct relationship between RIPK4 and EMT.

Our research revealed that RIPK4 can inhibit STAT3 pathway activation. Although the STAT3 pathway has been proven by many studies to be a key factor in many types of cancer (Xiu-Ping et al., 2018) (Attia et al., 2017) (Kopechek et al., 2015), there is only one study reporting that RIPK4 can significantly inhibit STAT3 signalling and inhibit cancer progression in lung adenocarcinoma 10. Matrix metalloproteinase-2 (MMP-2) and matrix metalloproteinase-9 (MMP-9) are enzymes related to degradation of the extracellular matrix and play an important role in the metastasis of tumours to distant tissues and organs. Related studies have proven that MMP-2 and MMP-9 exist downstream of STAT3 and play an important role in tumours (Kim et al., 2018) (Zou et al., 2015) (Huinan et al., 2018). Our research proves that RIPK4 can inhibit the phosphorylation of STAT3 in HCC cells, resulting in the downregulation of target gene matrix metalloproteinases (MMP-2 and MMP-9), thereby inhibiting the invasion and metastasis of HCC. This study provides many ideas for further research seeking to understand how RIPK4 regulates the STAT3 pathway.

## Conclusion

An increasing number of studies have shown that functional genes play an indispensable role in the occurrence and development of tumours. RIPK4 has been found to play different roles in various diseases. In our study, we verified that RIPK4 is downregulated in HCC tissues vs. normal tissues. We found that RIPK4 promotes tumour progression by promoting the invasion and metastasis of HCC and illustrated the close relationship between RIPK4 and the STAT3 pathway. RIPK4 also inhibits EMT, which is an important component of its inhibitory effects on HCC progression. Targeting both RIPK4 and the STAT3 pathway may be a new treatment strategy for HCC patients.

## Data Availability

The original contributions presented in the study are included in the article/Supplementary Material, further inquiries can be directed to the corresponding author.

## Abbreviations

AFP, *a*-fetoprotein; ALB, albumin; ALT, alanine aminotransferase; AST, aspartate aminotransferase; PLT, platelet; TBIL, total bilirubin.
