# Supplementary material for: RIPK4 Suppresses the Invasion and Metastasis of Hepatocellular Carcinoma by Inhibiting the Phosphorylation of STAT3
Source: Front Mol Biosci. 2021 Jun 18;8:654766. doi: 10.3389/fmolb.2021.654766 (PMC8249771; doi:10.3389/fmolb.2021.654766)
Supplement: Supplementary file 1 [file Table1.DOCX]

Supplementary Material

**Supplementary Table 1. Antibody information**

| **Antibodies** |  | |  | |  |  |
| --- | --- | --- | --- | --- | --- | --- |
| Name | Manufacturer | | Number | | dilution ratio | Usage |
| RIPK4 | Abnove | | H00054101-M05 | | 1:5000 | IHC |
| RIPK4 | Santa Cruz | | sc-100428 | | 1:1000 | WB |
| E-cadherin | Cell Signaling Technology | | #3195 | | 1:1000 | WB |
| N-cadherin | Cell Signaling Technology | | #13116 | | 1:1000 | WB |
| Zeb-1 | Cell Signaling Technology | | #3396 | | 1:1000 | WB |
| MMP-2 | Cell Signaling Technology | | #40994 | | 1:1000 | WB |
| MMP-9 | Cell Signaling Technology | | #13667 | | 1:1000 | WB |
| STAT3 | Cell Signaling Technology | | #9139 | | 1:1000 | WB |
| p- STAT3 | Cell Signaling Technology | | #9145 | | 1:1000 | WB |
| β-actin | Sigma-Aldrich | | A3854 | | 1:50000 | WB |
| goat anti-mouse IgG-HRP | absin | | abs20001 | | 1:8000 | WB |
| goat anti-rabbit IgG-HRP | absin | | abs20002 | | 1:8000 | WB |
| **Primer sequences (5’-3’)** | |  | |  | |  |
| RIPK4 | F: ATGCCCACTACCACGTCAAG R: AGGTAGGCGATTGTGCCAAA PCR | | | | | |
| GAPDH | F: TGACTTCAACAGCGACACCCA R: CACCCTGTTGCTGTAGCCAAA | | | | | |

**WB: Western blotting; IHC: Immunohistochemistry;**
